# Homœopathy and Blood-Letting

**Published:** 1891-01

**Authors:** 


HOMŒOPATHY AND BLOOD-LETTING. By W. B. Clarke, M.D., Secretary Indiana Institute of Homœopathy. Indianapolis. Reprinted from the *Medical Current* of November, 1890.

This pamphlet of fifteen pages is a most pungent exposure of the follies of blood-letting. After giving quotations from a review of a book on blood-letting, the writer makes this stinging remark:

"Travers may have 'eulogized' blood-letting, but history shows that Hahnemann did more—he embalmed it."

Dr. Gross, in his *Surgery*, marvels at the falling off of the practice of blood-letting, and predicts its restoration to favor at an early day. Such remarks seem very ludicrous to a homœopathist. Dr. Gross should have had a copy of Dr. Clarke's article to read. W. M. J.

